# An effective disease diagnostic model related to pyroptosis in ischemic cardiomyopathy

**DOI:** 10.1111/jcmm.17957

**Published:** 2023-09-19

**Authors:** Zhankui Jin, Fuqiang Liu, Guoan Zhang, Jingtao Zhang, Xiangrong Zhao, Xueping Huo, Xiaoyan Huang, Cuixiang Xu

**Affiliations:** ^1^ Department of Orthopedics Shaanxi Provincial People's Hospital Xi'an China; ^2^ Department of Cardiology Shaanxi Provincial People's Hospital Xi'an China; ^3^ Department of Cardiovascular Surgery Shaanxi Provincial People's Hospital Xi'an China; ^4^ Shaanxi Provincial Key Laboratory of Infection and Immune Diseases Shaanxi Provincial People's Hospital Xi'an China; ^5^ Shaanxi Engineering Research Center of Cell Immunology Shaanxi Provincial People's Hospital Xi'an China

**Keywords:** diagnosis, immune, ischemic cardiomyopathy, microenvironment, pyroptosis

## Abstract

Pyroptosis is involved in ischemic cardiomyopathy (ICM). The study aimed to investigate the pyroptosis‐related genes and clarify their diagnostic value in ICM. The bioinformatics method identified the differential pyroptosis genes between the normal control and ICM samples from online datasets. Then, protein–protein interaction (PPI) and function analysis were carried out to explore the function of these genes. Following, subtype analysis was performed using ConsensusClusterPlus, functions, immune score, stromal score, immune cell proportion and human leukocyte antigen (HLA) genes between subtypes were investigated. Moreover, optimal pyroptosis genes were selected using the least absolute shrinkage and selection operator (LASSO) analysis to construct a diagnostic model and evaluate its effectiveness using receiver operator characteristic (ROC) analysis. Twenty‐one differential expressed pyroptosis genes were identified, and these genes were related to immune and pyroptosis. Subtype analysis identified two obvious subtypes: sub‐1 and sub‐2. And LASSO identified 13 optimal genes used to construct the diagnostic model. The diagnostic model in ICM diagnosis with the area under ROC (AUC) was 0.965. Our results suggested that pyroptosis was tightly associated with ICM.

## 1 INTRODUCTION

Ischemic cardiomyopathy (ICM) typically includes a larger category of myocardial disease secondary to coronary artery disease.^1^ ICM involves multibranched coronary artery lesions or even diffuse disease resulting in extensive myocardial ischemia, necrosis and fibrosis, plus ischemic paralysis and hibernating myocardium. Therefore, ICM can lead to severe cardiac dysfunction or heart failure and eventually reduced survival.^2^, ^3^

ICM is a thorny problem in clinical treatment and is usually related to pyroptosis. Pyroptosis is a type of programmed cell death induced by caspase cleavage and gastrin protein (GSDMD or GSDME) activation.^4^ Pyroptosis exerts cellular inflammatory necrosis and accompany by cell lysis and inflammatory factors release like interleukin (IL)‐1β and IL‐18.^5^ Pyroptosis is closely related to various metabolic diseases and tumours.^6^, ^7^ Zeng et al. suggested that pyroptosis induced by NLRP3 activation through caspase‐1 promoted myocardial dysfunction progression.^8^ Furthermore, some studies have demonstrated that inhibiting GSDMD‐mediated pyroptosis in cardiomyocytes can effectively alleviate myocardial ischemia–reperfusion (IR) injury.^9^, ^10^ These pieces of evidence indicate that pyroptosis is involved in the progression of cardiomyopathy.

With the development of biology and informatics, bioinformatics was an essential tool to identify the potential diagnostic and prognosis biomarkers in numerous diseases. For example, Wu et al. found a potential pyroptosis‐related gene signature in atherosclerosis.^11^ The present study utilised bioinformatics analysis and identify pyroptosis‐related genes and explore the diagnostic value in ICM.

## 2 METHODS

### 2.1 Data source

GSE5406,^12^ GSE57338^13^ and GSE42955^14^ datasets were obtained from Gene Expression Omnibus (GEO, http://www.ncbi.nlm.nih.gov/geo/) database.^15^ GSE5406 contained 210 human cardiac tissue samples and 124 (108 ICM and 16 normal control) were involved in the analysis. GSE57338 contained 313 human cardiac tissue samples and 231 (95 ICM and 136 normal control) were involved in the analysis. GSE42955 contained 29 human cardiac tissue samples and 17 (12 ICM and 5 normal control) were involved in the analysis. The testing platform of GSE5406, GSE57338 and GSE42955 were GPL96 [HG‐U133A] Affymetrix Human Genome U133A Array, GP11532 [HuGene‐1_1‐st] Affymetrix Human Gene 1.1 ST Array and GP11532 [HuGene‐1_1‐st] Affymetrix Human Gene 1.1 ST Array, respectively. Data in GSE5406 and GSE57338 were generated into a training dataset after removing the batch using the sva package in R 3.6.1.^16^ Totally training dataset contained 203 ICM and 152 control samples. GSE42955 was considered the validating dataset.

### 2.2 Selection of differentially expressed pyroptosis genes

Forty‐seven pyroptosis genes were retrieved from a previous study.^17^ In the training dataset, intergroup *t*‐tests in R 3.6.1 were adopted to compare the differences between ICM and control groups. Genes with FDR less than 0.05 was considered the significantly differential expressed pyroptosis genes. The expression heat map of these differential expressed pyroptosis genes was visualised using heatmap in R 3.6.1.^18^, ^19^ Correlation between these genes was exerted using the cor function in R 3.6.1.

### 2.3 Protein–protein interaction (PPI) network and pathway analysis

STRING was used to explore the PPI of differentially expressed pyroptosis genes,^20^ and the network was visualised using Cytoscape.^21^ clusterProfiler package was adopted to explore the Gene Ontology (GO) and Kyoto Encyclopedia of Genes and Genomes (KEGG) pathways of the node in the PPI network. FDR less than 0.05 was set as the significant threshold.^22^

### 2.4 Subtype analysis based on differential expressed pyroptosis genes

ConsensusClusterPlus was used for a subtype analysis in the training dataset based on the differential expressed pyroptosis genes.^23^ Then, GSVA was used to evaluate the pyroptosis score of each sample in the training dataset, and the Kruskal‐Wallis test was used to compare the score differences between different subtypes.^24^

### 2.5 Correlation of subtype and immune feature

CIBERSORT was used to calculate the immune cell proportion,^25^ and the estimate package was used to calculate the estimate scores, immune scores, stromal scores and tumour purity.^26^ The Kruskal‐Wallis test was adopted to compare the different subtypes' immune cell proportions and scores. Moreover, human leukocyte antigen (HLA) gene expression between different subtypes was also compared by an intergroup *t*‐test.

### 2.6 KEGG enrichment analysis between subtypes

Based on the gene expression level in training datasets, GSEA was adopted to screen the significantly correlated pathways with subtypes.^27^ FDR less than 0.05 was considered to be significant.

### 2.7 Construction of diagnostic mode

Univariate logistic analysis was conducted to screen the significantly expressed pyroptosis genes with P less than 0.05 using the rms package.^28^ The Lars package's minor absolute shrinkage and selection operator (LASSO) algorithm were used to optimise the pyroptosis genes.^29^ In the training dataset, e1071 was used to construct a pyroptosis‐related diagnosis model (core: sigmoid kernel; cross: 100‐fold cross validation).^30^ The receiver operator characteristic (ROC) curve method in pROC package was used to analyse the diagnostic effectiveness of the diagnostic model in both training and validating datasets.^31^

### 2.8 Correlation between pyroptosis genes and immune feature

CIBERSORT was used to evaluate the proportion of immune cells in the training dataset. Differences in immune cell proportion between ICM and control groups were investigated using the Kruskal‐Wallis test.^32^ Furthermore, the expression of genes used to construct the diagnostic model and immune cell proportion was analysed using the cor function in R 3.6.1.

**FIGURE 1:**
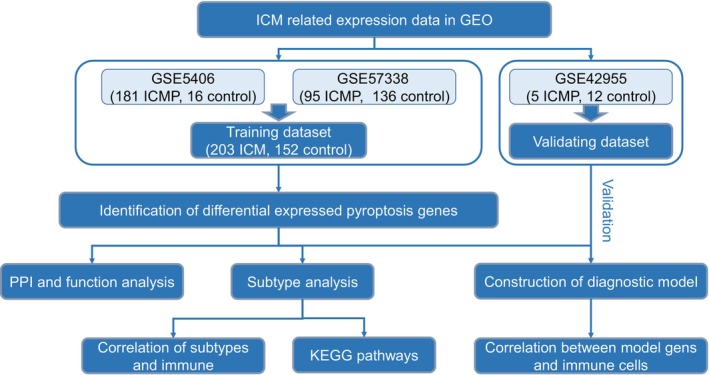
The study design. GEO, Gene Expression Omnibus; ICM, ischemic cardiomyopathy; PPI, protein–protein interaction; KEGG, Kyoto Encyclopedia of Genes and Genomes.

The study design was shown in Figure 1. We would determine the correlation between pyroptosis genes and ICM, investigate the differences of immune statues in different pyroptosis gene subtypes, and reveal the relation between pyroptosis genes and immune cells.

### 2.9 Real‐time quantitative PCR (RT‐qPCR)

A total of six myocardial samples, including three normal samples (control group; from donors without heart disease) and three ischemic cardiomyopathy samples (ICM group; from patients with ischemic cardiomyopathy) were collected from Shaanxi Provincial People's Hospital. The Ethical Committee of Shanxi Provincial People's Hospital approved this study, and the respective patient provided informed consent in written form. Total RNA extraction from myocardial samples was performed using Total RNA Kit I (Omega Bio‐tec, Inc.). Total RNA was converted into cDNA on the ice using the PrimeScript™ RT reagent kit (TaKaRa, Dalian, China). Quantitative PCR (qPCR) was performed using the Green PCR Master Mix System (Thermo Fisher Scientific). GAPDH was used as an internal reference. qPCR was carried out on an HT7900 real‐time PCR system (Applied Biosystems). The relative fold change was assessed with the $2^{-∆∆C_{T}}$ method. The primer sequences used are listed in Table 1.

**TABLE 1:** Primer sequences of real‐time PCR.

Gene	Primer sequences
Human‐BAX	F: 5′‐GATGGACGGGTCCGGG‐3′ R: 5′‐AGACACTCGCTCAGCTTCTT‐3′
Human‐CASP1	F: 5′‐AGGGAGGAGAGAAAAGCCAT‐3′ R: 5′‐GTCAATCAAAGCTCGGGTCT‐3′
Human‐CHMP2A	F: 5′‐TCATGATGGAGTTTGAGCGG‐3′ R: 5′‐GGAGGTTCGACAGCTCATC‐3′
Human‐GSDMB	F: 5′‐CCAAAGGGAAGTGACCATCC‐3′ R: 5′‐TCCTCTGTCAGGTCCTTGAG‐3′
Human‐GZMA	F: 5′‐GGGGCTCACTCAATAACCAG‐3′ R: 5′‐TGGTTCCTGGTTTCACATCG‐3′
Human‐GZMB	F: 5′‐ATCATCGGGGGACATGAGG‐3′ R: 5′‐GCCCCCAAGGTGACATTTAT‐3′
Human‐NLRP1	F: 5′‐TACTCCCCAAGGAACTGGAG‐3′ R: 5′‐GGATCAGAGTAGTTGCAGGC‐3′
Human‐NLRP3	F: 5′‐GTTCCTGAGGCTGGCATCT‐3′ R: 5′‐ACAGTTTACGGTGAACAACCA‐3′
Human‐NOD2	F: 5′‐AGCCTAATGGGCTTTGATGG‐3′ R: 5′‐CAGTCCAGGACACTCTCGAA‐3′
Human‐PYCARD	F: 5′‐CTCAGTCGGCAGCCAAG‐3′ R: 5′‐AGGCTGGTGTGAAACTGAAG‐3′
Human‐SCAF11	F: 5′‐CGACCTCGGTCTGAGGAAA‐3′ R: 5′‐CAGCCTCACTGTACAACAGA‐3′
Human‐TP53	F: 5′‐CAAAAGTCTAGAGCCACCGT‐3′ R: 5′‐TGTTTCCTGACTCAGAGGGG‐3′
Human‐TP63	F: 5′‐AGAACGGTGATGGTACGAAG‐3′ R: 5′‐GTACTGCATGAGTTCCAGGG‐3′
Human‐GAPDH	F: 5′‐ACCACAGTCCATGCCATCAC‐3′ R: 5′‐TCCACCACCCTGTTGCTGTA‐3′

### 2.10 Statistical analysis

All statistical analyses were conducted using R. A student's *t*‐test was used to compare AAA normal and ICM samples. ROC analysis was performed to estimate the discriminatory value of marker genes, with *p* < 0.05 considered to represent statistical significance.

## 3 RESULTS

### 3.1 Identification of differentially expressed pyroptosis genes

**FIGURE 2:**
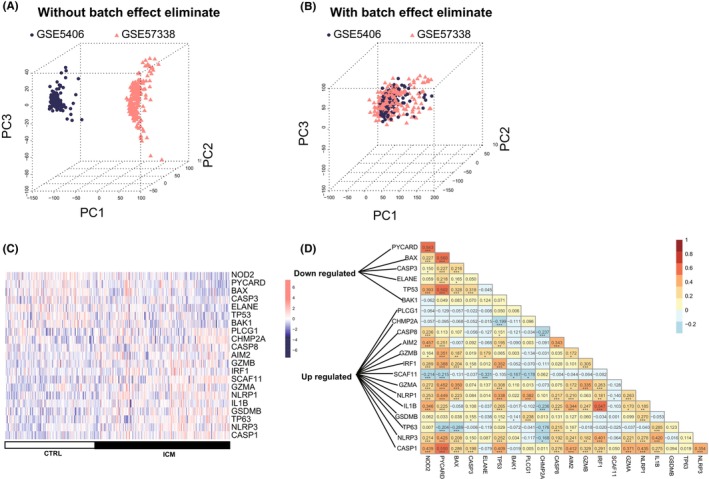
Selection of differentially expressed pyroptosis genes. (A) Samples before removing batch effect; (B) samples after removing batch effect. (C) Expression heat map of differentially expressed pyroptosis genes in ICM and control; (D) the correlation of differentially expressed pyroptosis genes.

We obtained the training dataset after combining the genes detected in GSE5406 and GSE57338 and removing the batch effects (Figure 2A,B). After a *t*‐test on 49 pyroptosis genes between ICM and control samples, 21 significantly expressed genes were selected. The expression pattern of the 21 genes in ICM and control groups was shown in Figure 2C. Correlation analysis showed that these genes were significantly correlated (Figure 2D).

### 3.2 PPI network and pathways analysis

**FIGURE 3:**
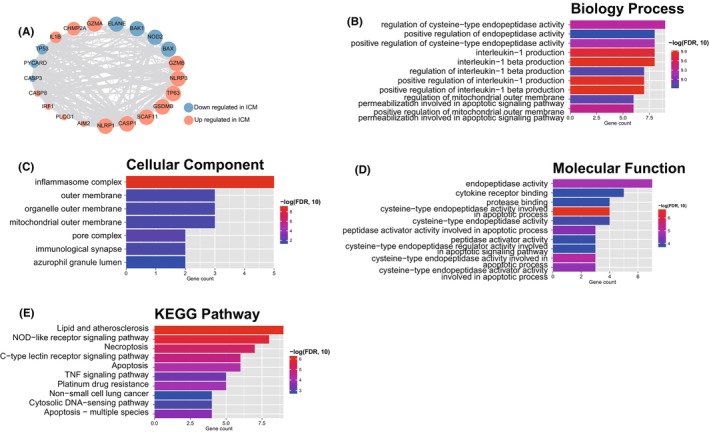
Protein–protein interaction (PPI) and function analysis. (A) PPI network of differentially expressed pyroptosis genes. Blue and orange represent the significantly downregulated and upregulated genes in ICM. The node's size presents significance, and larger means higher significance. The thickness of the connecting line indicates the interaction score, and the thicker the line, the higher the score. (B) Biology process of the differential expressed pyroptosis genes generated. (C) The cellular component of the differential expressed pyroptosis genes generated. (D) The molecular function of the differential expressed pyroptosis genes generated. (E) KEGG pathways of the differential expressed pyroptosis genes generated.

As represented in the methods, we searched the PPI related to 21 significantly expressed pyroptosis genes (Figure 3A). Further functional analysis showed that these genes were significantly correlated with 272 GO biology processes (BP), 7 GO cellular components (CC), 39 GO molecular functions (MF) and 21 KEGG pathways. The top 10 function were showed in Figure 3B–E. These results showed that these pyroptosis genes were related to immune response and pyroptosis process (like IL‐1β production).

### 3.3 Subtype analysis

**FIGURE 4:**
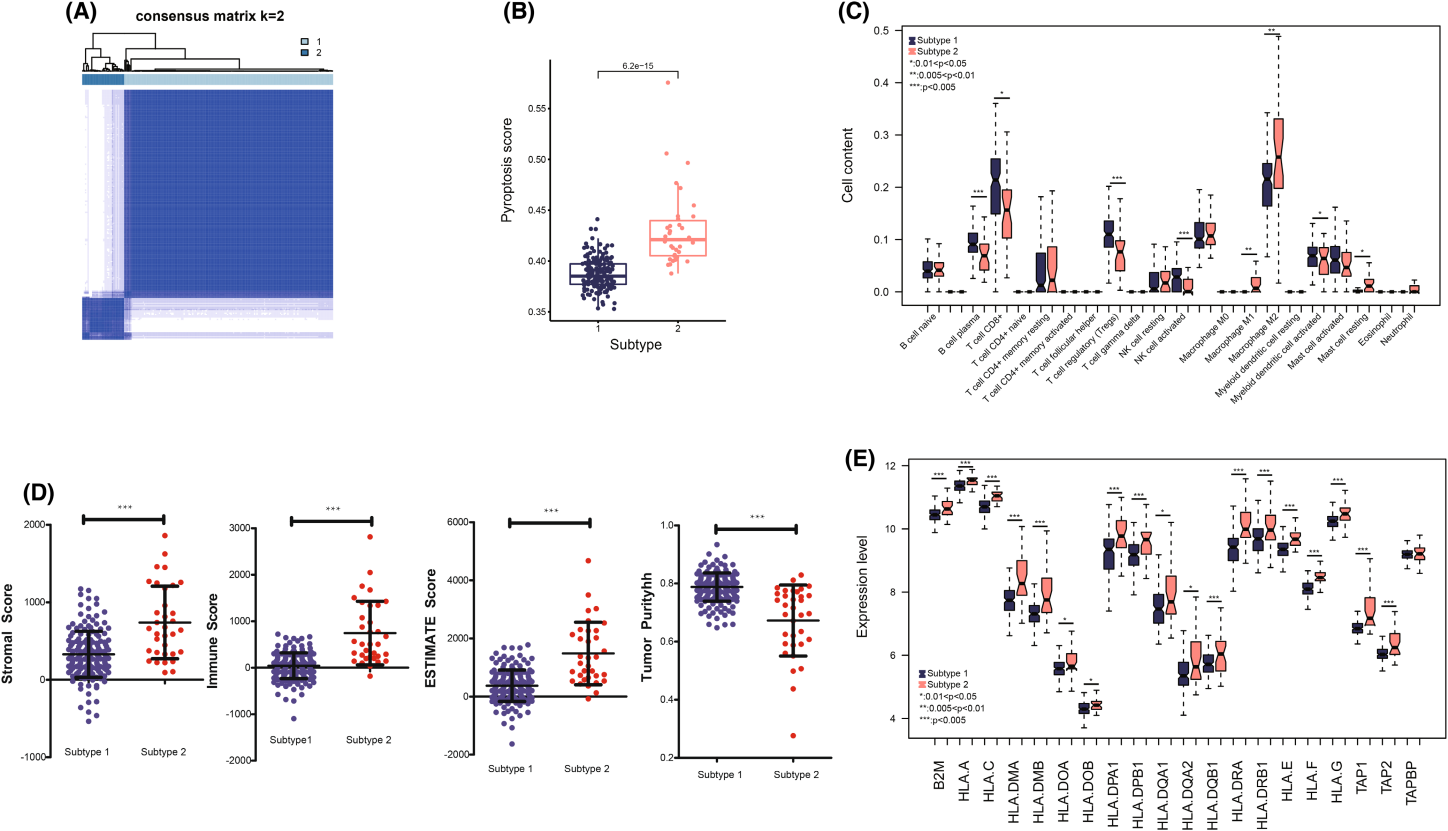
Subtype analysis. (A) All the samples in the training dataset are divided into sub‐1 and sub‐2. (B) sub‐2 exhibits a higher pyroptosis score. (C) Immune cells exhibit significantly different between sub‐1 and sub‐2. (D) Stromal, immune and estimate scores are higher in sub‐2 than in sub‐1, while tumour purity is lower in sub‐2 than in sub‐1. (E) Human leukocyte antigen (HLA) genes exhibit differences between sub‐1 and sub‐2.

Based on the 21 pyroptosis genes identified aforehand, all the ICM samples were classified into two obvious subtypes: sub‐1 and sub‐2 (Figure 4A), with 169 and 34 ICM samples, respectively. Further analysis showed that pyroptosis scores in sub‐2 were significantly higher than in sub‐1, demonstrating a more intense pyroptosis status (Figure 4B).

Considering the close correlation between pyroptosis and immune, we further investigated the differences in immune status in different subtypes. Eight types of immune cells were infiltrated significantly differently between the two subtypes (Figure 4C). For example, M2 macrophage was significantly higher in sub‐2 than in sub‐1. Stromal and immune scores in sub‐2 were significantly higher than in sub‐1, while tumour purity was significantly lower in sub‐2 than in sub‐1 (Figure 4D). Moreover, most of the HLA genes were significantly higher in sub‐2 than in sub‐1 (Figure 4E).

We also explored the function differences between the two subtypes. The results showed that 18 significantly different pathways existed between the two subtypes: apoptosis, chemokine signalling pathway and antigen processing and presentation (Figure S1).

### 3.4 Construction of a diagnostic model

**FIGURE 5:**
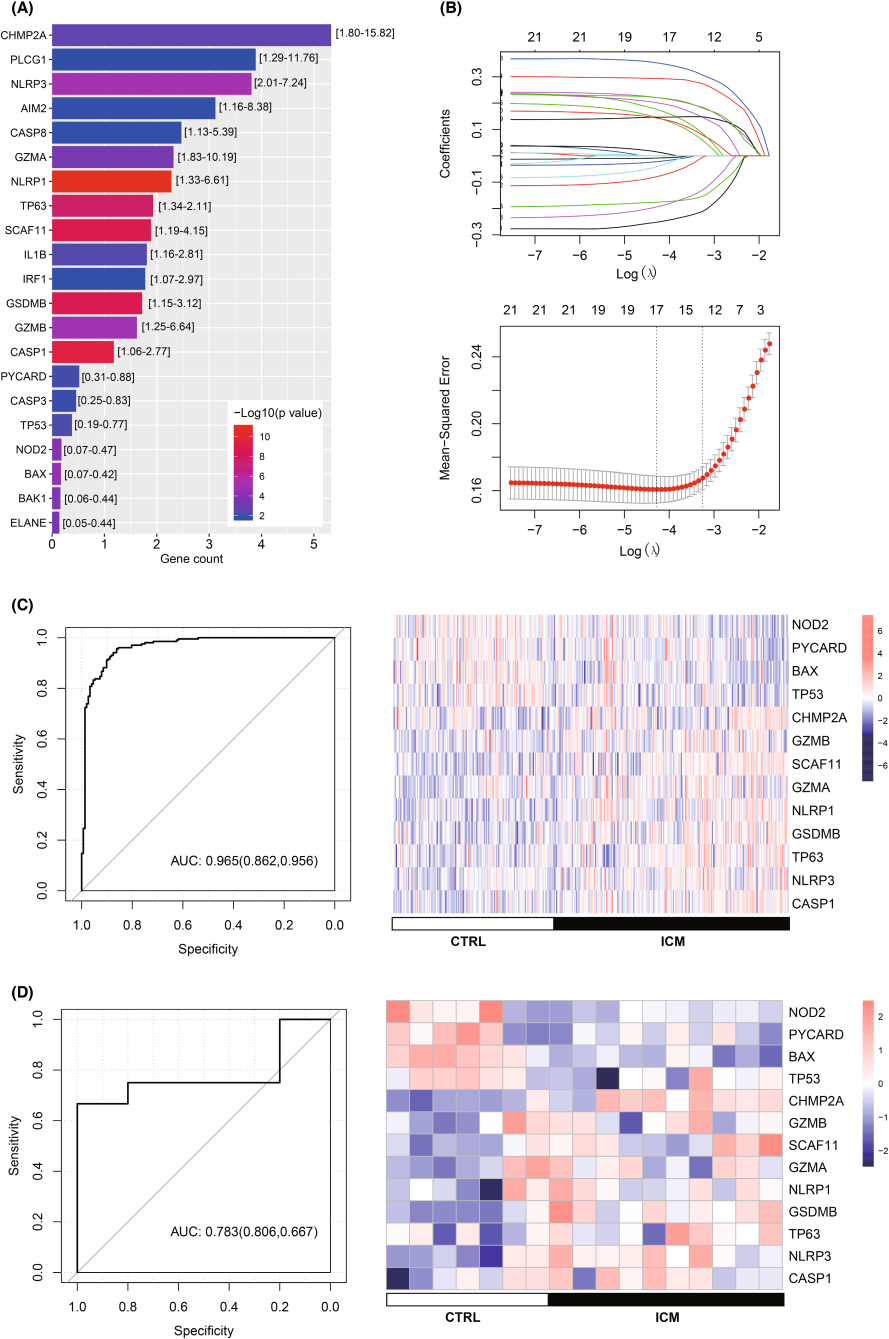
Construction of diagnostic model. (A) Optimal significantly differential expressed pyroptosis genes after univariate logistic analysis. (B) Most minor absolute shrinkage and selection operator (LASSO) analysis. Up is the distribution of the LASSO coefficient; down is the likelihood deviation of the LASSO coefficient distribution; the two vertical dashed lines represent lambda. min and Lambda.1SE, respectively. (C) ROC curve of the diagnostic model and expression of the 13 optimal pyroptosis genes in the training dataset. (D) Receiver operator characteristic (ROC) curve of the diagnostic model and expression of the 13 optimal pyroptosis genes in validating dataset.

Based on the 21 differential expressed pyroptosis genes, the univariate logistic analysis identified that all the 21 genes were significantly pyroptosis genes (Figure 5A, *p* < 0.05). Following LASSO analysis finally identified 13 optimal diagnosis genes: BAX, CASP1, CHMP2A, GSDMB, GZMA, GZMB, NLRP1, NLRP3, NOD2, PYCARD, SCAF11, TP53 and TP63 (Figure 5B). These optimal genes were generated to construct the diagnostic model based on data in the training dataset. ROC analysis showed that the model possessed perfect diagnostic effectiveness (Figure 5C left, AUC = 0.965). Also, the diagnostic value of the model was verified in validating dataset: the AUC was 0.783, demonstrating a promising diagnostic value (Figure 5D left). The expression level of these optimal genes exhibited different expression patterns in ICM and control samples (Figure 5C,D right).

### 3.5 Correlation of the pyroptosis genes and immune cell

**FIGURE 6:**
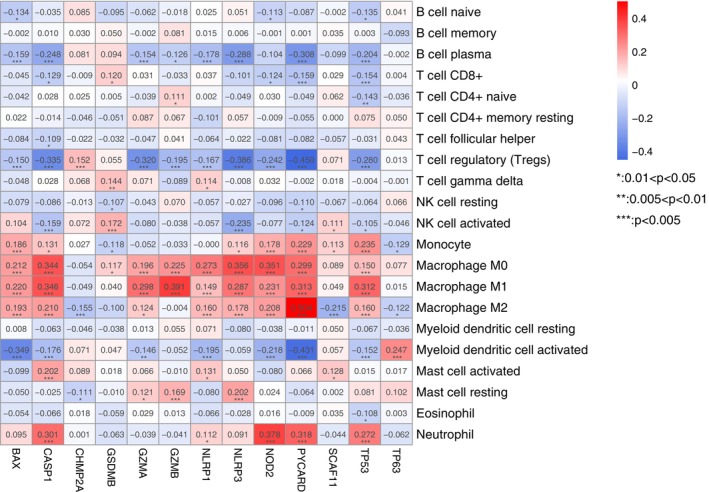
Correlation of the 13 optimal genes and immune cells proportion (*: 0.01 < *p* < 0.05; **: 0.005 < *p* < 0.01; ***: *p* < 0.005).

For further confirmed the performance of the optimal genes, we investigated the correlation of these genes and immune cell proportion. The results suggested that they were closely correlated (Figure 6). Except for CHMP2A, the expression levels of the other nine pyroptosis genes are closely positively correlated with M0 and M1 macrophages, especially CASP1, GZMA, GZMB, NLRP1, NLRP3, NOD2, PYCARD and TP3 (*p* < 0.005). CASP1, NOD2, PYCARD and TP3 are closely positively correlated with Neutrophils. However, ASP1, GZMA, GZMB, NLRP1, NLRP3, PYCARD and TP3 are tightly negatively associated with B cell plasma and T regulatory cells (Treg). PYCARD and TP3 are closely negatively correlated with CD8^+^ T cells (*p* < 0.005).

### 3.6 Q‐PCR validated the relationship of 13 pyroptosis gene levels between control and ICM

**FIGURE 7:**
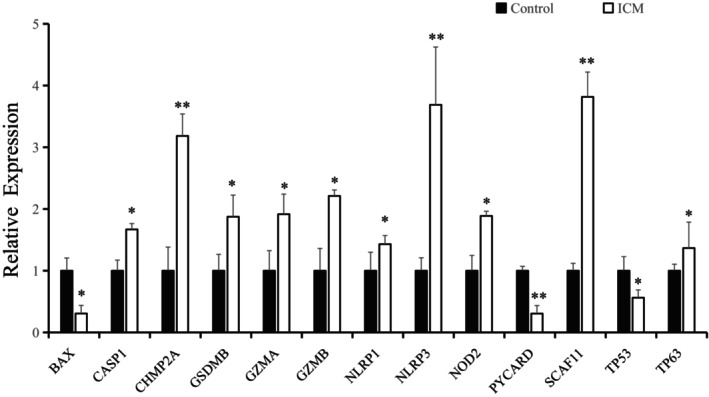
Verification of 13 optimal pyroptosis genes by qRT‐PCR in control and ICM group. Compared with control group, *p < 0.05, **p < 0.01.

RT‐qPCR results showed that compared with the control group, among the 13 optimal genes, the expression levels of 10 optimal genes (CASP1, CHMP2A, GSDMB, GZMA, GZMB, NLRP1, NLRP3, NOD2, SCAF11 and TP63) in ICM group were significantly upregulated (*p* < 0.05). The three optimal genes (BAX, PYCARD and TP53) in ICM group were significantly downregulated (*p* < 0.05). Among them, CHMP2A, NLRP3, PYCARD and SCAF11 showed the most significant differences between the two groups (*p* < 0.01) (Figure 7). The validation results confirmed the reliability of this diagnostic model.

## 4 DISCUSSION

Pyroptosis might be involved in the process of ICM. In the present study, we identified apparent subtypes and a diagnostic model which possessed promising diagnostic ability in ICM based on the pyroptosis‐related genes in ICM. The subtypes and the diagnostic model genes were significantly correlated with the immune status in ICM. These results possess guiding significance for future diagnosis and therapy in ICM.

The present study first identified 21 differential expressed pyroptosis‐related genes between control and ICM samples. Following functional analysis validated that these genes were associated with immune response and pyroptosis progression, like regulation of IL‐1β. Based on these 21 pyroptosis genes, all the samples were divided into two subtypes: sub‐1 and sub‐2. sub‐2 showed a higher pyroptosis score, higher immune cell infiltration level and higher HLA gene expression than sub‐1, indicating that sub‐2 exerted a more intense immune characteristic. A previous study has demonstrated that a change in macrophage phenotype may inhibit myocardial IR injury through various immune‐related pathways.^33^, ^34^ In our results, macrophage showed significant differences between the two subtypes, indicating a tight correlation between the subtypes classified by pyroptosis genes and immune cell infiltration. HLA is an essential component of the immune system;^35^ the differences of the HLA gene expression also demonstrate a tight correlation between subtypes and immune responses. In the future, we will further reveal the relationship and mechanism among pyroptosis genes in ICM suntypes, tumour purity, immune score and HLA.

We obtained 13 optimal pyroptosis genes in ICM from the univariate logistic and LASSO analysis. Based on the 13 pyroptosis genes, we constructed a diagnostic model for ICM. The model showed a perfect diagnostic ability, with AUC of 0.965 and 0.783 in training and validating datasets. The results demonstrate that the genes and the genes model may exhibit beneficial effects in diagnosing ICM.

As for the 13 optimal genes (BAX, CASP1, CHMP2A, GSDMB, GZMA, GZMB, NLRP1, NLRP3, NOD2, PYCARD, SCAF11, TP53 and TP63), their regulating role in ICM may be important, because their expression levels are different in ICM and control samples, and they are tightly correlated with immune cell levels. Chen et al. reveal that pyroptosis was involved in the pathogenesis of celiac disease, and γδ T cells exhibited high expression of IFN‐γ were the most relevant cells associated with pyroptosis.^36^ Previous studies have demonstrated the regulating role of these pyroptosis‐related genes. For example, BAX is the primary pro‐apoptosis gene, and dysregulation of BAX contributes to aberrant cell death.^37^ GSDMB cleaved by granzyme A from cytotoxic lymphocytes triggers pyroptosis in target cells.^38^ Moreover, GSDMB could trigger and regulate pyroptosis by alternative splicing killer lymphocytes.^38^ NLRP3 activation can induce pyroptosis through caspase‐1 and then promote myocardial dysfunction progression,^39^ and obesity‐associated protein inhibits NLRP3‐mediated pyroptosis to attenuate myocardial ischemia–reperfusion injury.^40^ Change of NLRP1 and CASP1 has also been demonstrated to be associated with cardiomyopathy.^41^ Silencing of NOD2 protects against diabetic cardiomyopathy in a murine diabetes model.^42^ Activation of the TP53‐related pathway can induce myocardial fibrosis, apoptosis, cardiac dysfunction and premature death.^43^ In the future, we will further determine the ICM treatment targets using the effective disease diagnostic model related to pyroptosis and reveal the relationship and mechanism between the targets and pyroptosis genes. We are, moreover, combining the effectiveness of the pyroptosis gene diagnostic model with the specificity of peripheral blood myocardial enzymes, immune cells and inflammatory factors to predict ischemic cardiomyopathy, which is expected to achieve early diagnosis and treatment.

The pyroptosis‐related genes play essential roles in the occurrence and development of cardiovascular disease and affect the process and outcomes. Inhibiting myocardial pyroptosis could improve cardiac remodelling.^44^ This study confirmed 13 optimal pyroptosis genes to be downregulated or upregulated in ICM, demonstrating a necessary diagnostic and regulating potential. We identified these genes as highly accurate biomarkers for the effective diagnosis of ICM enabling early treatment and intervention. If ICM is diagnosed early using this model, colchicine (inhibitors of NLRP3 inflammasome) can be used for treatment,^45^ significantly reducing infarct size and the expression levels of inflammatory markers.

Our results indicated an obvious subtype classification and effective diagnostic model based on the pyroptosis‐related genes. Nevertheless, our research has some limitations due to small sample sizes and a lack of databases available for analysis:

1. The diagnostic model's disease assessment and prediction accuracy can be improved by increasing the sample size.
2. The correlation between the subtypes, diagnostic genes and clinical features is absent, and further investigations should be conducted.
3. The relationship between the subtypes of pyroptosis genes and the progression of ICM, as well as their roles and mechanisms in the pathological process of ICM, will be further investigated.
4. The specific role of the most optimal genes in the ICM has yet to be elucidated.

Therefore, in the future, in the follow‐up study, we will continue to excavate the specific mechanism of pyroptosis‐related genes in the progression of ischemic cardiomyopathy and clarify the correlation between pyroptosis‐related genes and ICM progression.

In summary, our results suggested that pyroptosis was tightly associated with ICM. Pyroptosis‐related genes could be used as ICM diagnostic biomarkers. The different expression patterns of these pyroptosis genes would provide more information for ICM diagnosis and treatment.

## AUTHOR CONTRIBUTIONS

**Zhankui Jin:** Data curation (lead); formal analysis (equal); methodology (lead); software (lead); writing – original draft (lead). **Fuqiang Liu:** Formal analysis (equal); methodology (supporting); writing – original draft (equal). **Guoan Zhang:** Methodology (supporting); resources (equal); writing – original draft (equal). **Jingtao Zhang:** Methodology (supporting); resources (equal). **Xiangrong Zhao:** Methodology (supporting); validation (equal). **Xueping Huo:** Methodology (equal); validation (equal). **Xiaoyan Huang:** Funding acquisition (lead); methodology (supporting); project administration (lead); writing – original draft (lead); writing – review and editing (lead). **Cuixiang Xu:** Conceptualization (lead); funding acquisition (lead); project administration (lead); writing – original draft (equal).

## FUNDING INFORMATION

Open Project of the Key Laboratory for the Prevention and Treatment of Cardiovascular Diseases in Shaanxi Province with Integrated Traditional Chinese and Western Medicine in Shaanxi University of Traditional Chinese Medicine, Grant/Award Number: 2022XXG‐QN‐001; the Key Projects of Shaanxi Provincial Department of Education, Grant/Award Number: 22JS035; Science and Technology Incubation Fund Program of Shaanxi Provincial People's Hospital, Grant/Award Number: 2022YJY‐59.

## CONFLICT OF INTEREST STATEMENT

The authors declare no conflict of interest.

## Supporting information


Figure S1.

## Data Availability

The data supporting this study's findings are available from the corresponding author upon reasonable request.
