# Supplementary figures and images for: An effective disease diagnostic model related to pyroptosis in ischemic cardiomyopathy
Source: J Cell Mol Med. 2023 Sep 19;27(23):3816–26. doi: 10.1111/jcmm.17957 (PMC10718138; doi:10.1111/jcmm.17957)

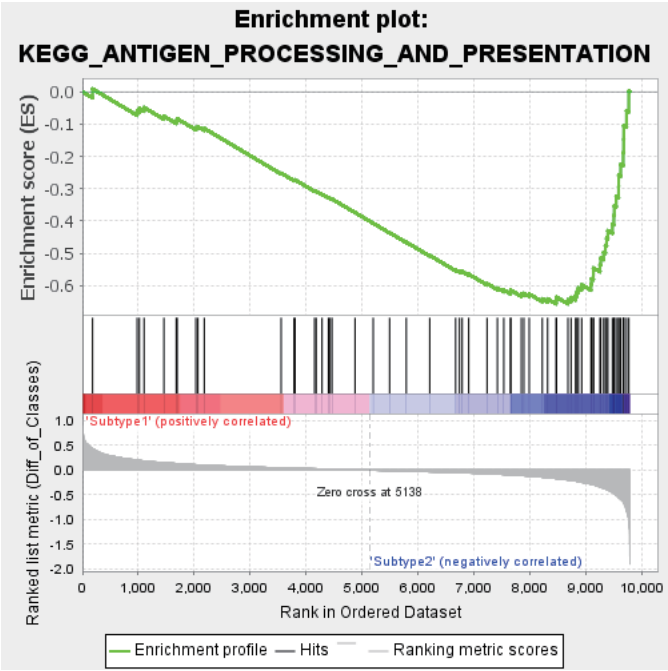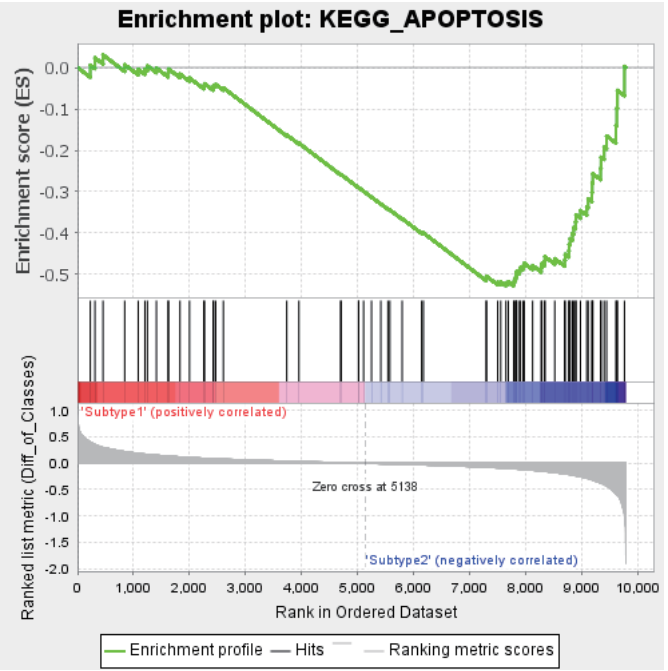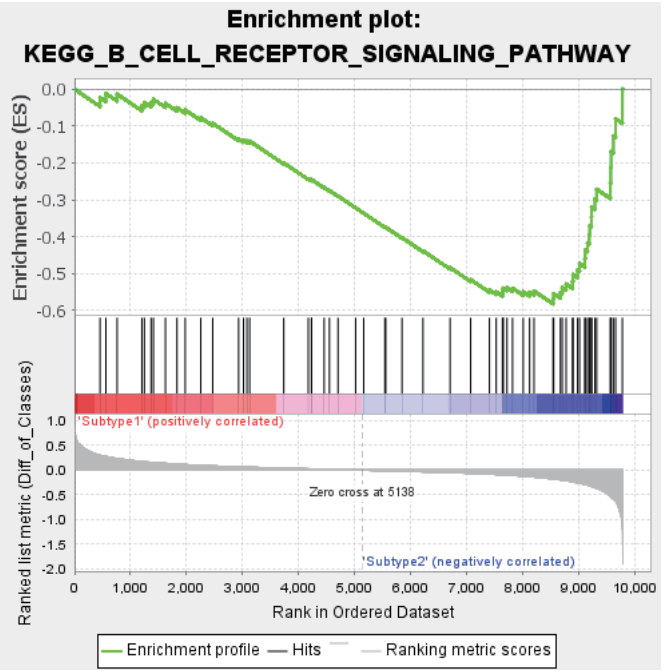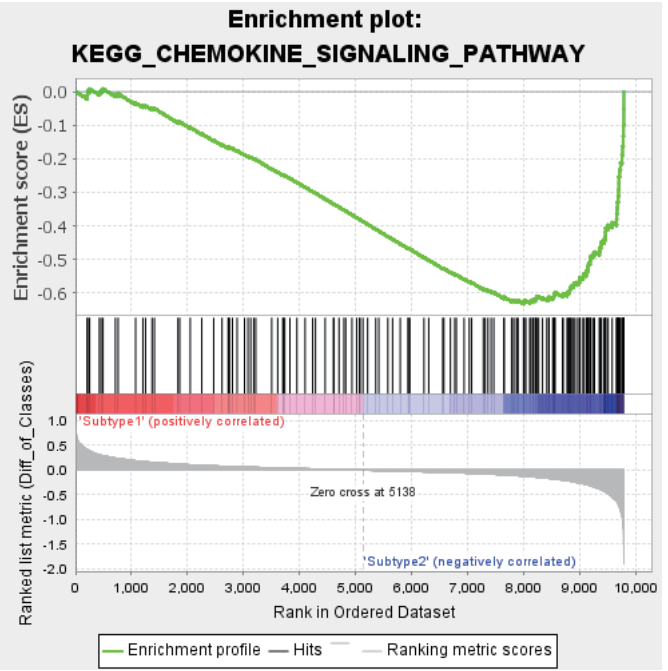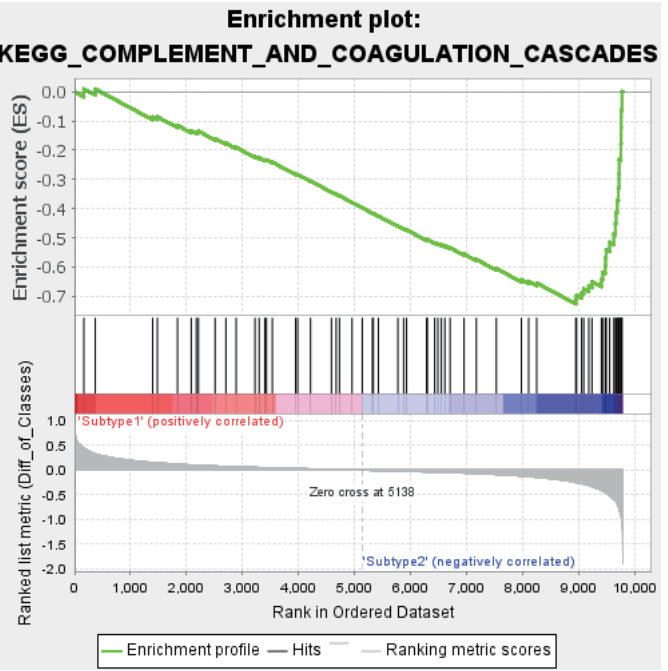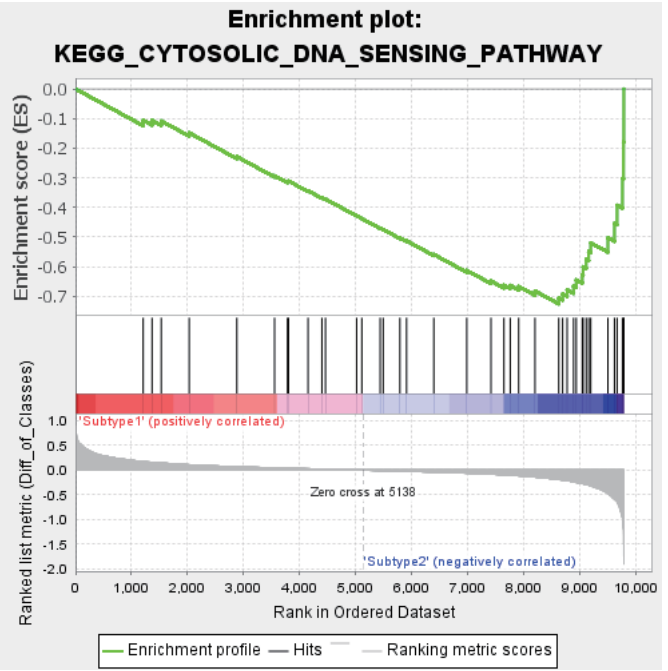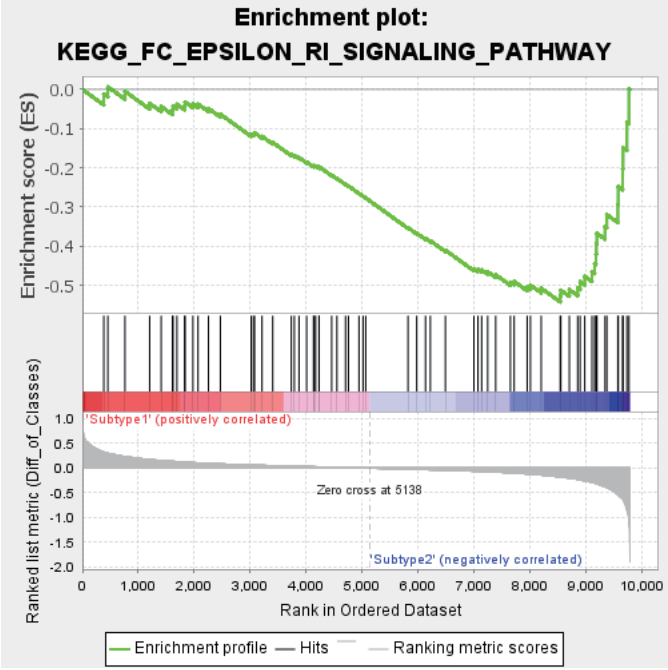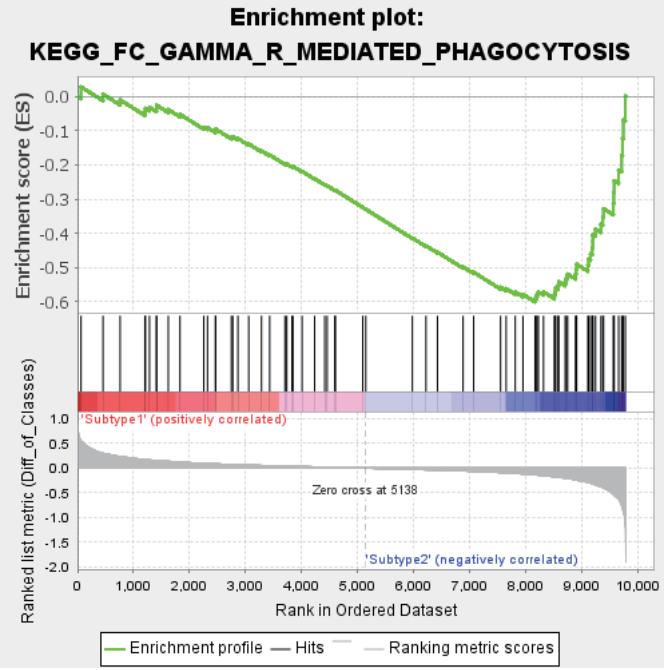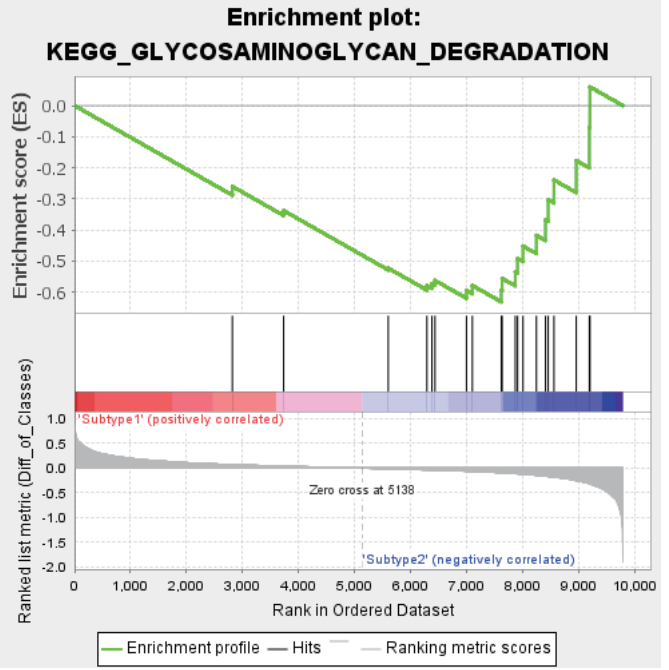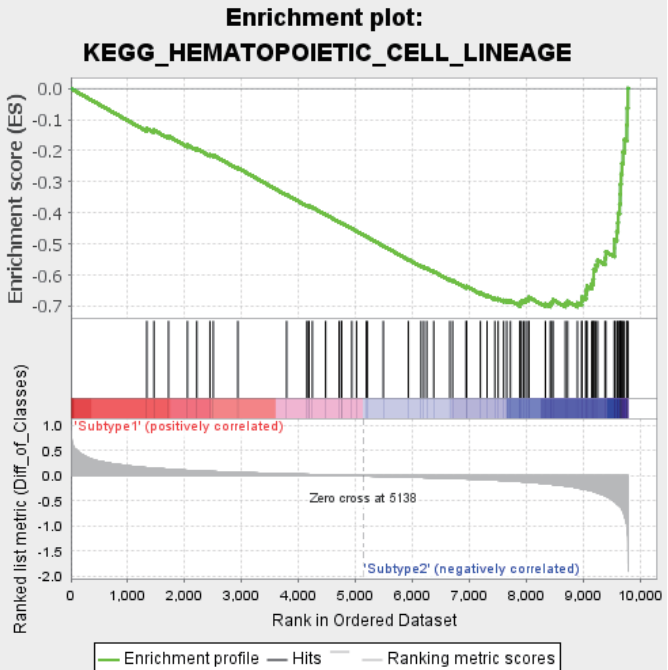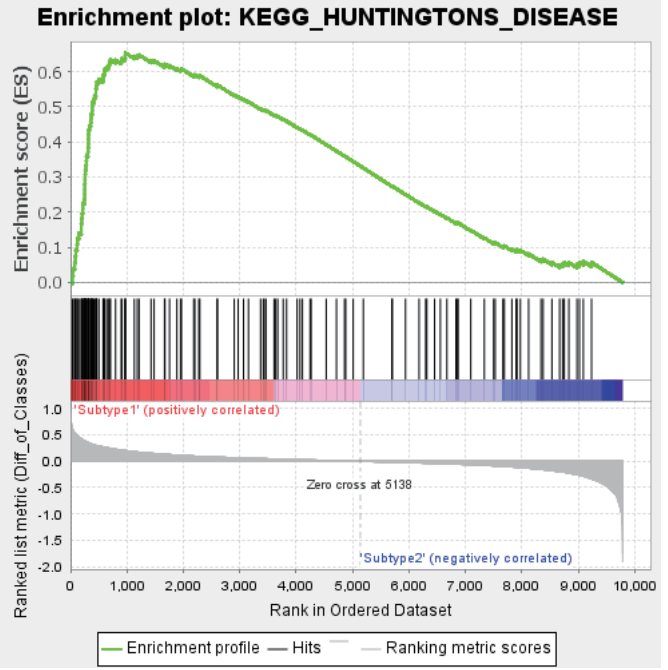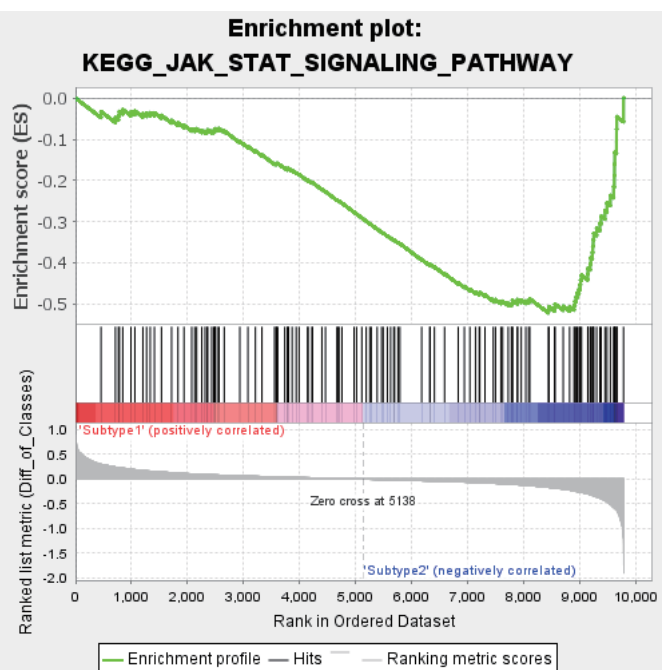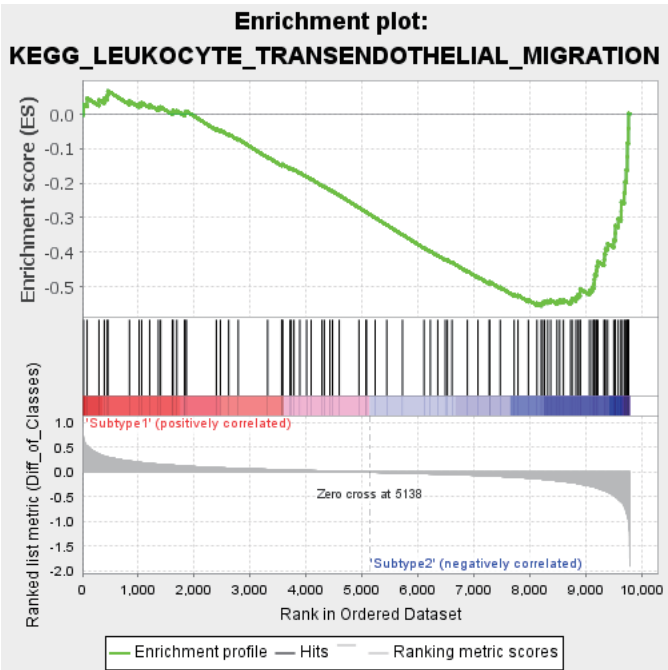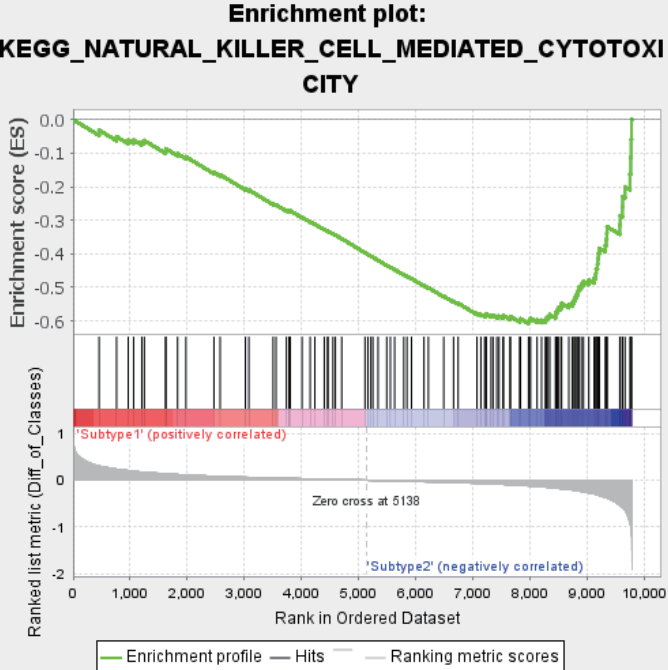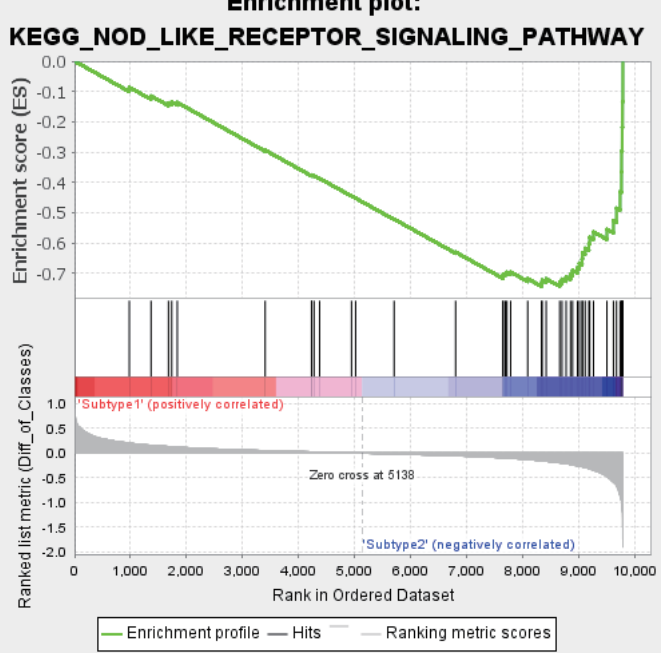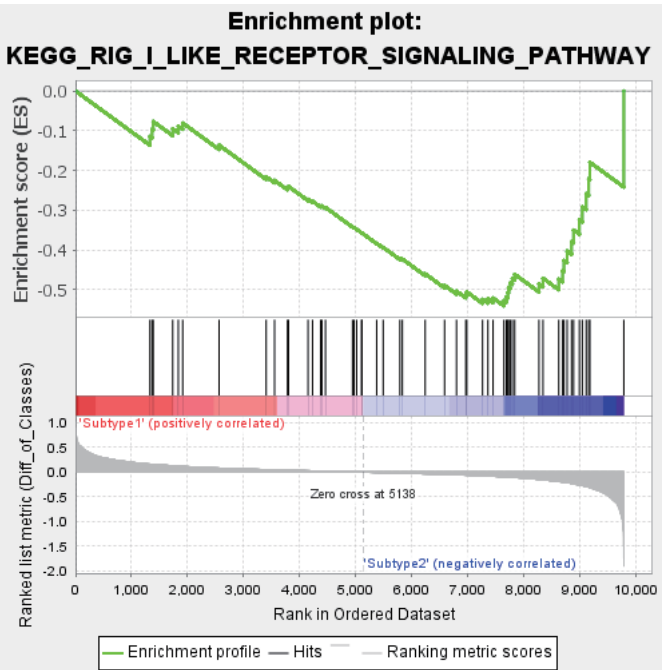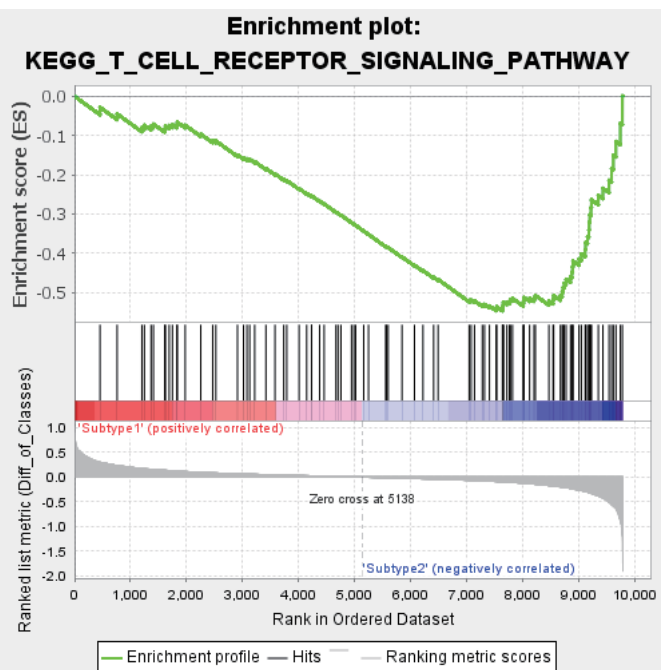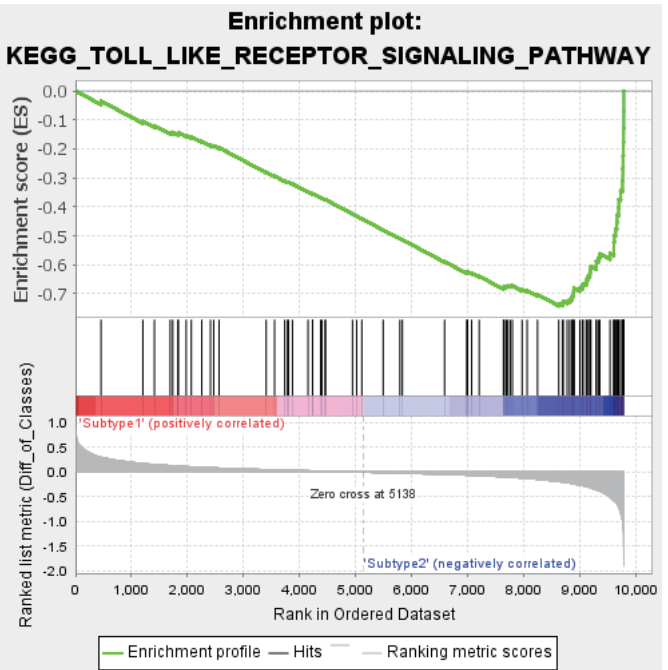

Supplement: Supplementary file 1 — Figure S1. [file JCMM-27-3816-s001.pdf]
